# High-Temperature Deformation Behavior of Powder Metallurgy Ti-4Zr-6Al-0.6Si-0.5Mo Alloy

**DOI:** 10.3390/ma19010117

**Published:** 2025-12-29

**Authors:** Zongshu Li, Wentao Liu, Jian Wang, Yuankui Cao, Qihong Fang, Ao Fu, Bin Liu

**Affiliations:** 1 China North Nuclear Fuel Co., Ltd., Baotou 014035, China; lizongshu0909@163.com (Z.L.); liuwentao0506@163.com (W.L.); 2 State Key Laboratory of Powder Metallurgy, Central South University, Changsha 410083, China; 213307006@csu.edu.cn (J.W.); caoyuankui@csu.edu.cn (Y.C.); binliu@csu.edu.cn (B.L.); 3 State Key Laboratory of Advanced Design and Manufacturing Technology for Vehicle, College of Mechanical and Vehicle Engineering, Hunan University, Changsha 410082, China; fangqh1327@hnu.edu.cn

**Keywords:** titanium alloy, high-temperature deformation, constitutive equation, processing map

## Abstract

The hot deformation behavior of Ti-4Zr-6Al-0.6Si-0.5Mo high-temperature titanium alloy was investigated under temperatures of 800–1100 °C, strain rates of 0.001–1 s^−1^, and a true strain of 0.5. Flow stress curves under different temperatures and strain rates were analyzed, and a constitutive equation that depicts the correlation among flow stress, deformation temperature, and strain rate was established. Processing maps were employed for determining the instability domains as well as optimal hot processing window. The results show that the optimal hot processing window for the alloy is a deformation temperature of 950–1100 °C and a strain rate of 0.001–0.01 s^−1^. Flow instability occurred within two domains: 800–850 °C at strain rates of 0.1–1 s^−1^ and 900–1075 °C at strain rates of 0.01–0.1 s^−1^, where the alloy is prone to cracking, resulting in processing failure.

## 1. Introduction

Near-α high-temperature titanium alloys represent a prominent development trend within the realm of titanium alloys over the past decade, which synergistically combine low density with high strength, enabling structural weight reduction while preserving load-bearing stability [1,2,3,4]. Furthermore, they exhibit reliable high-temperature mechanical properties, rendering them adaptable to complex and demanding service conditions [5,6,7,8]. As a result, they have been widely adopted in critical industries—including aerospace, shipbuilding, and the chemical industry—providing a robust material basis for the development of lightweight, high-performance equipment and underscoring their significant application value and development potential [9,10,11,12].

Hot working is an essential process in manufacturing metal components, which not only imparts the desired shape and dimensions but also further enhances the overall properties of the alloy [13,14,15]. Compared with steels and aluminum alloys, titanium alloys are often more difficult to hot work due to their unique crystal structure [16,17]. During hot working, deformation temperature and strain rate represent the two most vital parameters, requiring precise control to ensure uniform deformation [15,18,19]. Thermomechanical simulation experiments are capable of efficiently simulating the deformation characteristics under various temperatures and strain rates, reproducing the material’s response during actual hot working [20,21]. Processing maps founded on the dynamic materials model (DMM) are employed to evaluate and predict material workability, visually delineating the safe and instability domains [22,23,24]. However, investigations into the microstructural evolution and deformation behavior of near- α high-temperature titanium alloys are rarely reported.

In this research, the hot deformation behavior of a powder metallurgy Ti-4Zr-6Al-0.6Si-0.5Mo alloy is investigated. By analyzing stress-strain curves, characterizing microstructures, and constructing processing maps, the deformation mechanisms under various deformation conditions were elucidated, aiming to offer a reference for the optimization of the hot working process of this alloy.

## 2. Experimental Procedure

**Figure 1:**
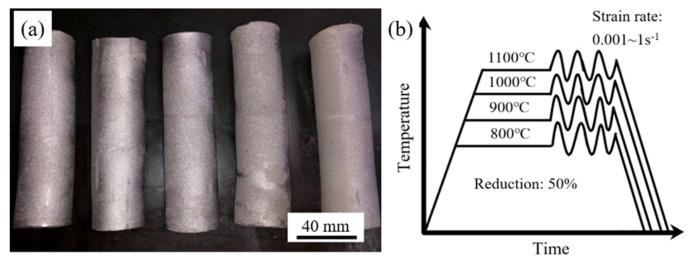
(**a**) Ti-4Zr-6Al-0.6Si-0.5Mo sintered rods and (**b**) procedure of hot compression tests.

Elemental Ti, Zr, Al, Si, and Mo powders (99.99 wt.%, TIJO, Changsha, China) were mixed in a V-type mixer for 5 h under an argon atmosphere and subsequently subjected to cold isostatic pressing at 200 MPa for 5 min. The green compacts (porosity: 22%) were thereafter sintered in a high-vacuum furnace (JK-320T, LANLING, Shenyang, China) at 1300 °C for 4 h and then furnace-cooled to 25 °C, thus obtaining sintered rods (Figure 1a). Table 1 presents the chemical composition, which matches the designed composition.

Cylindrical compression specimens with a diameter of 8 mm and a height of 10 mm were machined from the sintered rods (relative density: 96%) using wire electrical discharge. High-temperature compression experiments were conducted using a Gleeble-3180 machine (Dynamic System Inc., Poestenkill, NY, USA) in vacuum within a temperature interval of 800–1100 °C at steps of 100 °C. The strain rate values are 0.001, 0.01, 0.1, and 1 s^−1^. To guarantee a homogeneous temperature profile, the samples were heated to the target temperature at 3 °C/s and held for 3 min. All samples were subjected to a deformation corresponding to a 50% height reduction and then immediately subjected to water quenching to room temperature. Each compression experiment was repeated three times. The hot compression process is schematically depicted in Figure 1b. The compressed samples were sectioned along the compression direction and observed with a Quanta FEG 250 scanning electron microscope (SEM, FEI, Hillsboro, OR, USA) for microstructural analysis. The samples for SEM were fabricated via wire electrical discharge machining, sequentially ground with 400-, 800-, and 2000-grit abrasive papers, and finally polished using colloidal silica suspension. Phase structure characterization was performed via an X-ray diffractometer (XRD, D/max-2550, Rigaku Corporation, Akishima, Tokyo, Japan) equipped with Cu Kα radiation. Differential scanning calorimetry (DSC) measurements were carried out using a simultaneous thermal analyzer (NETZSCH STA 449F3, NETZSCH, Selb, Bavaria, Germany) under argon atmosphere, with a heating rate of 10 K/min from ambient temperature to 1200 °C.

## 3. Results and Discussion

### 3.1. Initial Microstructure

**Figure 2:**
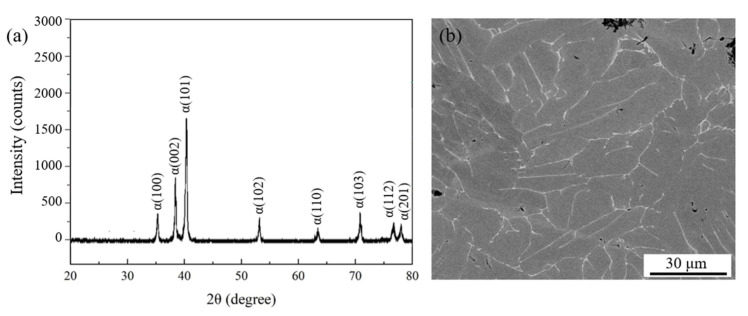
(**a**) XRD pattern and (**b**) SEM image of the Ti-4Zr-6Al-0.6Si-0.5Mo alloy.

Figure 2a presents the XRD pattern of the Ti-4Zr-6Al-0.6Si-0.5Mo alloy. Characteristic peaks of the α phase are observed, confirming its classification as a near-α titanium alloy. Meanwhile, as shown in SEM image, gray α phase, bright β phase, and residual microporosity (~4 vol.%, Figure 2b) were identified as the main microstructural features of the alloy. The β phase is primarily distributed between elongated α-phase lamellae. Quantification of Figure 2b indicated a β-phase content of 2.8%, which is below the detection limit of XRD, thus accounting for the absence of β-phase diffraction peaks in Figure 2a.

**Figure 3:**
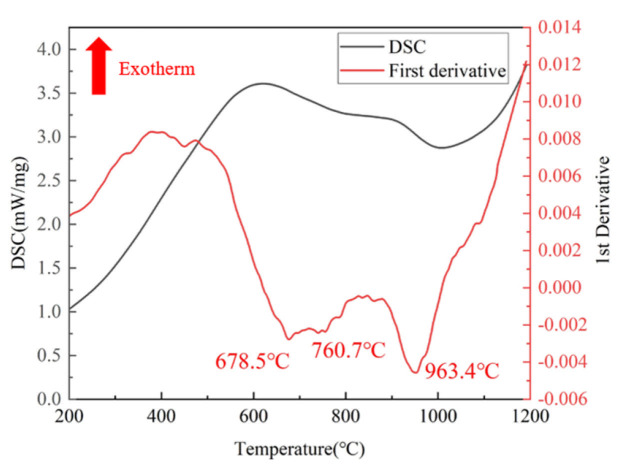
DSC curve of the Ti-4Zr-6Al-0.6Si-0.5Mo alloy.

Figure 3 shows the DSC curve of the Ti-4Zr-6Al-0.6Si-0.5Mo alloy. Three endothermic peaks are observed between 200 °C and 1200 °C. The first two endothermic peaks are related to the ω-phase transformation commonly found in titanium alloys, while the third peak corresponds to the alloy’s phase transformation [26,27,28]. To accurately determine the phase transformation temperature, the first derivative of the original curve was obtained, yielding the red curve. The temperature at which the third peak appears is clearly identified as 963.4 °C. Therefore, the compression temperature was set as 800–1100 °C for the subsequent research of the high-temperature deformation behavior of the Ti-4Zr-6Al-0.6Si-0.5Mo alloy.

### 3.2. Flow Behavior

**Figure 4:**
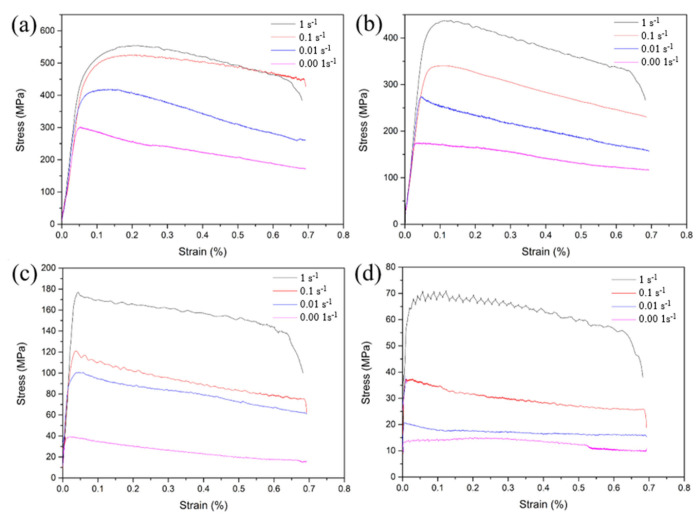
True stress–true strain curves of the Ti-4Zr-6Al-0.6Si-0.5Mo alloy under different deformation temperatures: (**a**) 800 °C, (**b**) 900 °C, (**c**) 1000 °C, (**d**) 1100 °C.

The hot deformation characteristics of Ti-4Zr-6Al-0.6Si-0.5Mo alloy were evaluated via true stress–true strain curves (Figure 4) obtained at temperatures ranging from 800 to 1100 °C and strain rates of 0.001–1 s^−1^. All tests were conducted to a final true strain of 0.5, with flow stress data presented in Table 2. During initial hot working, the alloy exhibits rapid work hardening, where flow stress rises to a peak value at the corresponding peak strain. Work hardening dominates this stage. As deformation proceeds, the flow stress gradually decreases, indicating pronounced flow softening. Subsequently, work hardening and flow softening attain a dynamic equilibrium, thereby allowing the flow stress to stabilize. These observations can be attributed to: (1) In the initial deformation stage, a significant increase in dislocation density and mutual hindrance among tangled dislocations gives rise to work hardening, which in turn leads to a swift elevation of flow stress. (2) Beyond the peak stress, enhanced dynamic recrystallization, dynamic recovery, and dynamic α-phase spheroidization contribute to flow softening, causing a stress decrease. (3) At elevated strain levels, the flow stress maintains relative stability due to the dynamic balance between work hardening and flow softening [29,30,31].

### 3.3. Constitutive Analysis

**Figure 5:**
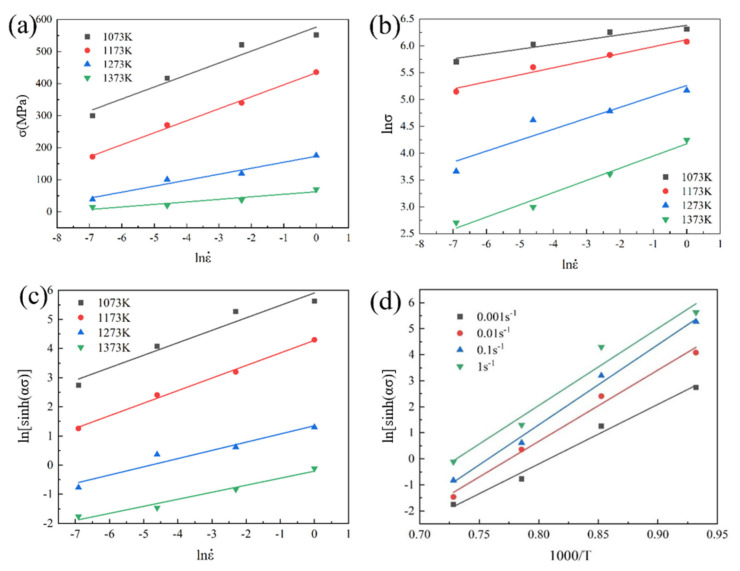
Relationship of peak compressive stress to strain rate and deformation temperature for Ti-4Zr-6Al-0.6Si-0.5Mo alloy: (**a**) σ-ln$\dot{\varepsilon }$, (**b**) lnσ-ln$\dot{\varepsilon }$, (**c**) ln[sinh(ασ)]-ln$\dot{\varepsilon }$, (**d**) ln[sinh(ασ)]-1000/T.

Generally, flow stress–temperature–strain rate relationships during hot deformation are typically modeled using the Arrhenius constitutive equation [32,33]:

(1) $$Z=\dot{\varepsilon }exp\left(\frac{Q}{RT}\right)=A_{1}\sigma^{n_{1}}(\alpha \sigma <0.8)$$

(2) $$Z=\dot{\varepsilon }exp\left(\frac{Q}{RT}\right)=A_{2}exp(\beta \sigma )(\alpha \sigma >1.2)$$

(3) $$Z=\dot{\varepsilon }exp\left(\frac{Q}{RT}\right)=A[sinh(\alpha \sigma ){]}^{n}(\mathrm{for}\;\mathrm{all}\;\sigma )$$

where Z (Zener–Hollomon parameter) = f($\dot{\varepsilon }$, σ, Q); R = 8.314 J·mol^−1^·K^−1^ (gas constant); T = deformation temperature; α, β, A_1_, A_2_, A = material constants; and n = β/α (stress exponent). Equations (1)–(2): low-/high-stress regimes; Equation (3): all stress levels. Logarithmic expressions:

(4) $$ln\;\dot{\varepsilon }=ln\;A_{1}+n_{1}ln\;\sigma -\frac{Q}{RT}$$

(5) $$ln\;\dot{\varepsilon }=ln\;A_{2}+\beta \sigma -\frac{Q}{RT}$$

(6) $$ln\;\dot{\varepsilon }=ln\;A+nln\;[sinh(\alpha \sigma )]-\frac{Q}{RT}$$

where $n_{1}=(\partial ln\;\dot{\varepsilon }/\partial ln\;\sigma {)}_{T}$, $\beta =(\partial ln\;\dot{\varepsilon }/\partial \sigma {)}_{T}$, and $n=\left\{\partial ln\;\dot{\varepsilon }/\partial ln\;[sinh(\alpha \sigma ){\}}_{T}\right.$ correspond to the slopes of linear curves ln$\dot{\varepsilon }$-lnσ, ln$\dot{\varepsilon }$-σ, and ln$\dot{\varepsilon }$-ln[sinh(ασ)], respectively. Substituting the peak stress of the Ti-4Zr-6Al-0.6Si-0.5Mo alloy at different strain rates as the characteristic parameter into Equations (4)–(6) yields the linear curves ln$\dot{\varepsilon }$-lnσ, ln$\dot{\varepsilon }$-σ, and ln$\dot{\varepsilon }$-ln[sinh(ασ)] at 800, 900, 1000, and 1100 °C (Figure 5a–c). The values for α = β/n_1_ and n were computed to be 0.011446 and 3.088501, respectively.

The activation energy of the Ti-4Zr-6Al-0.6Si-0.5Mo alloy can be calculated using the following equation [34]:

(7) $$Q=R{\left\{\frac{\partial ln\;\dot{\varepsilon }}{\partial ln\;[sinh(\alpha \sigma )]}\right\}}_{T}{\left\{\frac{\partial ln\;[sinh(\alpha \sigma )]}{\partial (1/T)}\right\}}_{\dot{\varepsilon }}$$

where ${\left\{\frac{\partial ln\;[sinh(\alpha \sigma )]}{\partial (1/T)}\right\}}_{\dot{\varepsilon }}$ corresponds to the slope of the ln[sinh(ασ)] versus 1000/T plot. By substituting the peak stress values of the Ti-4Zr-6Al-0.6Si-0.5Mo alloy at different temperatures as the characteristic parameter into Equation (6), linear ln[sinh(ασ)]-1/T curves can be constructed for strain rates of 0.001, 0.01, 0.1, and 1 s^−1^ (Figure 5d). The activation energy Q was computed as 396.9329 ± 15.6 kJ/mol. Additionally, the parameter A = 3.759765 × 10^18^ was determined from the intercept of the ln[sinh(ασ)]-1000/T linear curves.

**Figure 6:**
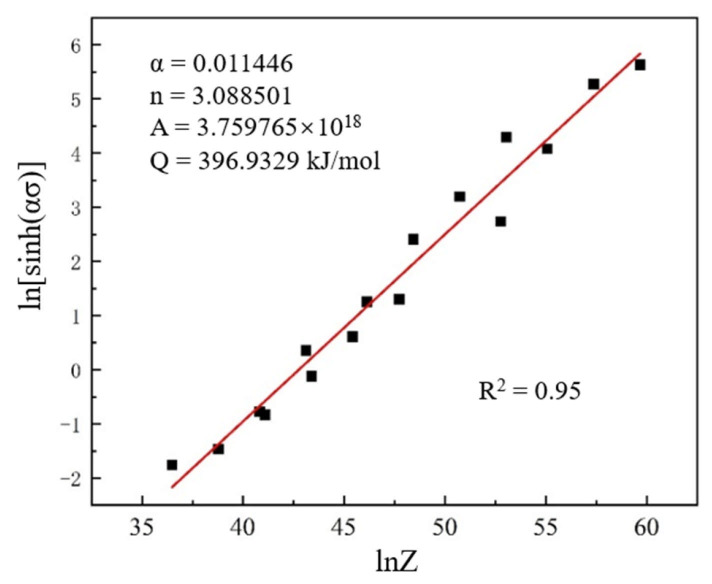
Relationship of peak compressive stress to Z parameter for Ti-4Zr-6Al-0.6Si-0.5Mo alloy.

In summary, the material constants for Ti-4Zr-6Al-0.6Si-0.5Mo alloy during hot compression are α = 0.011446, n = 3.088501, A = 3.759765 × 10^18^, and Q = 396.9329 kJ/mol. Substituting these constants into Equation (3) yields the Z values corresponding to different deformation conditions. By plotting the linear ln[sinh(ασ)]-lnZ curve, the value of R^2^ = 95% was obtained, indicating that the flow stress constitutive equation established by Equation (3) exhibits high accuracy (Figure 6). Therefore, the flow stress equation for Ti-4Zr-6Al-0.6Si-0.5Mo alloy during hot compression is expressed as follows:

(8) $$\dot{\varepsilon }=3.759765\times 10^{18}\times [sinh(0.011446\sigma ){]}^{3.088501}\times e^{\frac{-396.9329}{RT}}$$

### 3.4. Processing Maps

The evaluation of intrinsic workability via processing maps enables identification of optimal processing parameters, microstructural changes during deformation, and conditions for plastic instability. According to DMM theory, the total power P consumed during hot working partitions into two components [35]:

(9) $$P=\sigma \dot{\varepsilon }=G+J=\int_{0}^{\dot{\varepsilon }}\;\sigma d\dot{\varepsilon }+\int_{0}^{\sigma }\;\dot{\varepsilon }d\sigma$$

Here, G is the energy dissipated during plastic deformation, J represents the dissipative co-content for microstructural evolution, and $\dot{\varepsilon }$ and σ denote strain rate and flow stress. The power dissipation efficiency (η) is defined as the fraction of total energy consumed by microstructural evolution, and its mathematical expression is given by

(10) $$\eta =\frac{2m}{2m+1}$$

where $m=\frac{dJ}{dG}={\left[\frac{\partial (ln\;\sigma )}{\partial (ln\;\dot{\varepsilon })}\right]}_{\varepsilon ,T}$ represents the strain-rate sensitivity [36]. The power dissipation efficiency bears a close correlation with microstructural evolution during hot deformation. A higher power dissipation efficiency indicates that energy consumption for microstructural changes during hot working is highly efficient, with more extensive dynamic recovery and dynamic recrystallization, leading to better workability of the alloy [37,38,39,40,41].

Furthermore, voids and cracks represent common instability defects that may emerge during alloy hot working. The equation below is employed to evaluate such occurrences [42]:

(11) $$\xi (\dot{\varepsilon })=\frac{\partial (ln\;(m/m+1))}{\partial (ln\;\dot{\varepsilon })}+m<0$$

Here, $\xi (\dot{\varepsilon })$ is termed the instability factor. When $\xi \left(\dot{\varepsilon }\right)<0$, it indicates the emergence of flow instability. Equations (10) and (11), combined with strain, strain rate, and deformation temperature, allow construction of the alloy’s power dissipation and instability maps. The hot processing map is derived from the superposition of the power dissipation map and the instability map.

**Figure 7:**
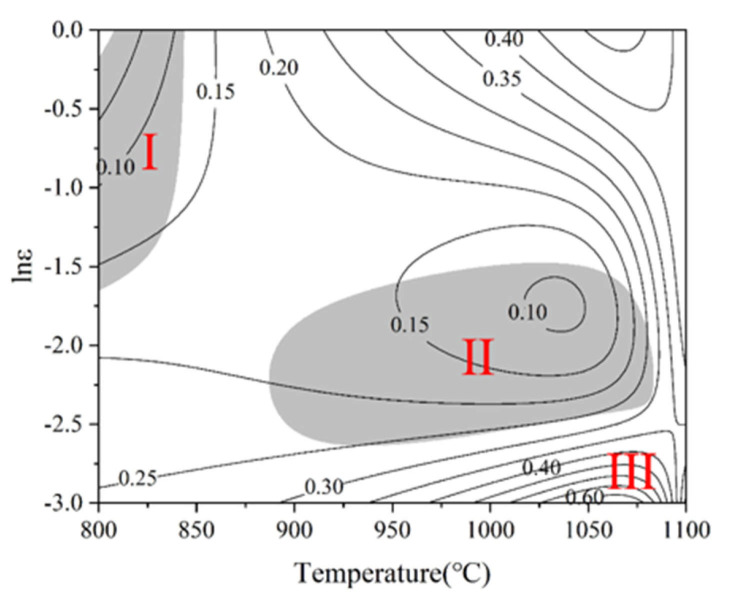
Hot processing map of Ti-4Zr-6Al-0.6Si-0.5Mo alloy at a strain of 0.5.

Figure 7 shows the hot processing map of the Ti-4Zr-6Al-0.6Si-0.5Mo alloy at 0.5 strain. In Figure 7, the black solid lines represent contours of constant power dissipation efficiency, while the gray areas indicate instability domains. Within instability domains, a large proportion of the externally imparted energy is consumed by plastic deformation, leading to macroscopic cracking and rendering these conditions unsuitable for hot working. In the stable processing region, higher power dissipation efficiency promotes dynamic recrystallization, resulting in flow softening and eventual steady-state flow. The map reveals that the Ti-4Zr-6Al-0.6Si-0.5Mo alloy exhibits two instability domains, labeled I and II. Domain I spans deformation temperatures of 800–850 °C and 0.1–1 s^−1^ strain rates, whereas Domain II covers temperatures of 900–1075 °C and 0.01–0.1 s^−1^ strain rates. These domains are prone to the formation of unstable microstructures, including flow localization, adiabatic shear bands, voids, and cracks. The regions outside the shaded areas represent stable deformation domains, among which Domain III (950–1100 °C, 0.001–0.01 s^−1^) with higher power dissipation efficiency (0.4 < η < 0.6) exhibits the best workability. This specific zone can be regarded as the most favorable hot working parameter range for the Ti-4Zr-6Al-0.6Si-0.5Mo alloy.

### 3.5. Hot Deformation Microstructure

**Figure 8:**
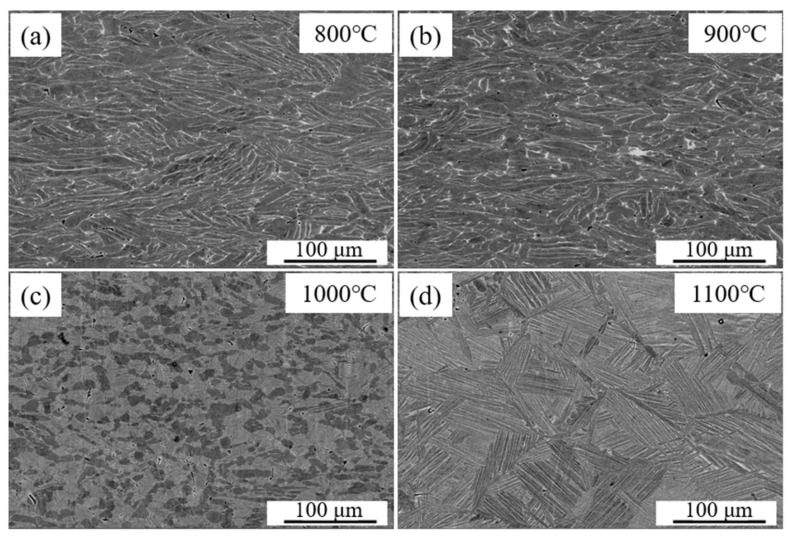
Deformed microstructures of Ti-4Zr-6Al-0.6Si-0.5Mo alloy at 0.001 s^−1^ under different temperatures: (**a**) 800 °C, (**b**) 900 °C, (**c**) 1000 °C, (**d**) 1100 °C.

Figure 8 shows the microstructures of the Ti-4Zr-6Al-0.6Si-0.5Mo alloy after deformation at 0.001 s^−1^ strain rate and temperatures of 800, 900, 1000, and 1100 °C. Micrographs reveal that the deformed alloy is a dual-phase titanium alloy comprising α phase (dark gray contrast) and β phase (light gray contrast), with residual porosity essentially eliminated. At 800 °C and 900 °C, the microstructure exhibits a kinked lamellar structure (Figure 8a,b). As the temperature increased to 1000 °C, pronounced α-phase spheroidization occurred, transforming the initial lamellar α phase into equiaxed or short rod-shaped α particles. Consequently, 1100 °C deformation in the β monophase range yielded total conversion to β phase; upon cooling, the original β grain boundaries disappeared, and lamellar α phase precipitated within the grains, yielding a basketweave microstructure. No adiabatic shear bands or localized deformation bands were observed in any of the microstructures deformed at the four temperatures, indicating that at this relatively low strain rate (0.001 s^−1^), hot working is performed across the entire temperature range without causing stress concentrations that lead to surface or internal defects.

**Figure 9:**
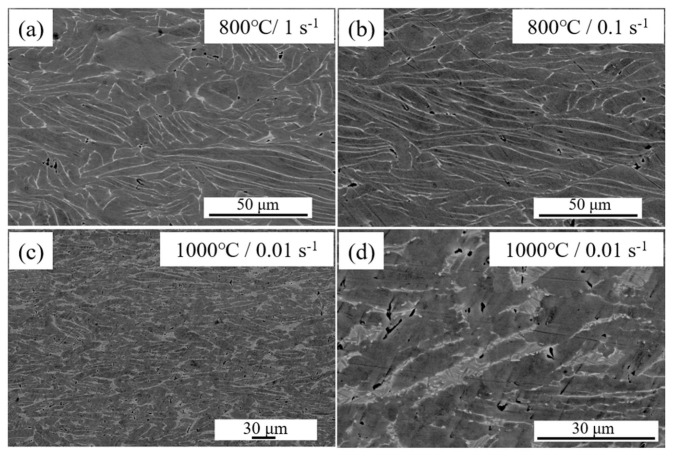
Microstructure of the Ti-4Zr-6Al-0.6Si-0.5Mo alloy deformed in the flow instability area: (**a**) 800 °C, 0.1s^−1^, (**b**) 800 °C, 0.01s^−1^ corresponding to area I, (**c**,**d**) 1000 °C, 0.01s^−1^ corresponding to area II.

Figure 9 shows the microstructures of the Ti-4Zr-6Al-0.6Si-0.5Mo alloy after deformation in the instability domains. Figure 9a,b present the microstructures after deformation in instability domain I (800 °C, 1 s^−1^ and 800 °C, 0.1 s^−1^). The figures reveal that the alloy microstructure exhibits distortion, accompanied by the formation of fine void defects after hot deformation at 800 °C. Processing in this domain is prone to cracking, leading to processing failure, and is unsuitable for hot working. Figure 9c,d show the microstructures after deformation in instability domain II (1000 °C, 0.01 s^−1^). Hot deformation in this domain also resulted in numerous defects, indicating that this region is likewise unsuitable for hot working.

## 4. Conclusions

In this work, Ti-4Zr-6Al-0.6Si-0.5Mo titanium alloy was fabricated via powder metallurgy. Its hot deformation behavior under conditions of 800–1100 °C and 0.001–1 s^−1^ was systematically analyzed. The main conclusions are:

1. Ti-4Zr-6Al-0.6Si-0.5Mo alloy exhibits typical near-α titanium alloy microstructure, accompanied by a minor quantity of retained β phase located between α-phase lamellae.
2. The alloy displays pronounced flow softening during high-temperature compression. The material’s flow stress is reduced by increasing temperature but enhanced by elevated strain rates.
3. A processing map was constructed at 0.5 strain. Ideal hot processing occurs at 950–1100 °C with strain rates of 0.001–0.01 s^−1^. Flow instability occurred in two domains: 800–850 °C at 0.1–1 s^−1^ and 900–1075 °C at 0.01–0.1 s^−1^, where cracking is prone to occur, leading to processing failure and rendering these regions unsuitable for hot working.
4. The deformation mechanism of Ti-4Zr-6Al-0.6Si-0.5Mo alloy at low temperatures (800–900 °C) is primarily localized flow or kinking, whereas at higher temperatures (1000–1100 °C), the dominant mechanisms are dynamic α-phase spheroidization and dynamic recrystallization. Microcracks emerged in high strain-rate zones, with crack density diminishing as strain rates decreased.

## Figures and Tables

**Table 1:** Chemical composition of the Ti-4Zr-6Al-0.6Si-0.5Mo alloy (wt.%) [25].

Elements	Ti	Zr	Al	Mo	Si	C	N	O
Fraction	Bal.	4.05	5.95	0.48	0.62	0.02	0.01	0.23

**Table 2:** Flow stress under different temperatures and strain rates.

Temperature (°C)	Strain Rate (s^−1^)	Flow Stress (MPa)
800	0.001	301.4 ± 16.8
	0.01	415.4 ± 12.3
	0.1	518.7 ± 20.1
	1	546.5 ± 25.6
900	0.001	172.0 ± 9.1
	0.01	268.1 ± 12.4
	0.1	339.1 ± 18.2
	1	435.1 ± 26.7
1000	0.001	39.1 ± 3.5
	0.01	99.3 ± 5.2
	0.1	119.9 ± 4.3
	1	172.9 ± 7.7
1100	0.001	13.5 ± 2.1
	0.01	20.2 ± 1.8
	0.1	37.3 ± 3.4
	1	65.1 ± 4.9

## Data Availability

The original contributions presented in this study are included in the article. Further inquiries can be directed to the corresponding author.
